# Effects of Physical Activity and Inactivity on Microvasculature in Children: The Hong Kong Children Eye Study

**DOI:** 10.1167/iovs.65.14.7

**Published:** 2024-12-03

**Authors:** Xiu Juan Zhang, Vincent L. Yuen, Yuzhou Zhang, Ka Wai Kam, Jason Wong, Fang Yao Tang, Alvin Young, Patrick Ip, Li Jia Chen, Tien Y. Wong, Chi Pui Pang, Clement C. Tham, Carol Y. Cheung, Jason C. Yam

**Affiliations:** 1 Department of Ophthalmology and Visual Sciences, The Chinese University of Hong Kong, Hong Kong SAR, China; 2 Department of Ophthalmology, University of Hong Kong, Hong Kong SAR, China; 3 Department of Ophthalmology and Visual Sciences, Prince of Wales Hospital, Hong Kong SAR, China; 4 Department of Paediatrics and Adolescent Medicine, University of Hong Kong, Hong Kong SAR, China; 5 Singapore Eye Research Institute, Singapore National Eye Center, Singapore; 6 Hong Kong Eye Hospital, Kowloon, Hong Kong SAR, China; 7 Department of Ophthalmology, Hong Kong Children's Hospital, Hong Kong SAR, China; 8 Hong Kong Hub of Paediatric Excellence, The Chinese University of Hong Kong, Hong Kong SAR, China; 9 Tsinghua Medicine, Beijing Tsinghua Changgung Hospital, Tsinghua University, Beijing, China

**Keywords:** physical activity, paediatric ophthalmology, retinal vasculature, deep learning

## Abstract

**Purpose:**

The purpose of this study was to investigate the effects of physical activity and inactivity on the microvasculature in children, as measured from retinal photographs.

**Methods:**

All participants were from the Hong Kong Children Eye Study, a population-based cross-sectional study of children aged 6 to 8 years. They received comprehensive ophthalmic examinations and retinal photography. Their demographics and involvement in physical activity and inactivity were obtained from validated questionnaires. A validated Deep Learning System was used to measure, from retinal photographs, central retinal arteriolar equivalent (CRAE) and central retinal venular equivalent (CRVE).

**Results:**

In the final analysis of 11,959 participants, 6244 (52.2%) were boys and the mean age was 7.55 (1.05) years. Increased ratio of physical activity to inactivity was associated with wider CRAE (*β* = 1.033, *P* = 0.007) and narrower CRVE (*β* = –2.079, *P* < 0.001). In the subgroup analysis of boys, increased ratio of physical activity to inactivity was associated with wider CRAE (*β* = 1.364, *P* = 0.013) and narrower CRVE (*β* = –2.563, *P* = 0.001). The subgroup analysis of girls also showed increased ratio of physical activity to inactivity was associated with narrower CRVE (*β* = –1.759, *P* = 0.020), but not CRAE.

**Conclusions:**

Increased activity in children is associated with healthier microvasculature, as shown in the retina. Our study contributes to the growing evidence that physical activity positively influences vascular health from a young age. Therefore, this study also underscores the potential of using the retinal vasculature as a biomarker of cardiovascular health.

Physical activity improves cardiovascular health in children,^1^ which can be carried forward into adulthood.^2^ However, the 2019 to 2020 National Survey of Children's Health showed that only 20.6% of 6- to 17-year-old children in the United States achieved the suggested activity duration from the Physical Activity Guidelines for Americans.^3^ Physical inactivity, on the other hand, can lead to being overweight and developing obesity, which increases the risk of developing cardiovascular diseases in childhood as well as in adulthood.^4^^–^^6^

Physical activity and inactivity and such other cardiovascular risk factors as diabetes and tobacco smoking have been shown to affect the microvasculature, including in the myocardium, skin, and retina.^7^^–^^9^ Previous studies suggested that increased physical activity was associated with angiogenesis in the myocardium and with greater skin vasodilation.^7^^,^^10^ In the retina, changes in the retinal vessel caliber have been associated with not only retinal pathologies, such as diabetic retinopathy, glaucoma, and age-related macular degeneration,^11^^–^^13^ but also cardiovascular diseases, such as hypertension, diabetes mellitus, and coronary heart disease.^14^^–^^16^ Therefore, the retinal vasculature has been suggested as a biomarker of cardiovascular health for predicting the risk of cardiovascular diseases.^17^^,^^18^ In fact, adding an analysis of the retinal vasculature may imply reclassifying 21% and 10% of subjects for risks of cardiovascular diseases and stroke, respectively.^19^^,^^20^

Some previous studies suggested that changes in the microvasculature, that is, narrower arterioles and wider venules, may imply cardiovascular risk factors, such as higher blood pressure and obesity,^21^ and, therefore, can be tracked to long-term end-organ damage and mortality of cardiovascular diseases.^22^ Whereas previous studies in adults have shown that increased physical activity is associated with narrower retinal venular caliber,^23^^–^^25^ there are few studies showing the effects of physical activity and inactivity on the microvasculature in children.^26^^,^^27^ Particularly, validated and noninvasive measures of the microvasculature in children are not widely available to address this knowledge gap.

In this study, we utilized a newly developed and validated artificial intelligence (AI) Deep Learning System to measure retinal arteriolar and venular caliber in children,^28^ and investigated the effects of physical activity and inactivity on the retinal vasculature. Our primary hypothesis is that increased activity would be associated with healthier retinal vasculature, as characterized by wider arterioles and narrower venules.

## Materials and Methods

### Study Population

Participants were recruited from the ongoing Hong Kong Children Eye Study (HKCES), a population-based cross-sectional study of eye conditions among schoolchildren aged 6 to 8 years.^29^^–^^31^ All children were invited to visit the Chinese University of Hong Kong Eye Centre for comprehensive ocular examinations and to submit standardized questionnaires according to a unified protocol.^29^ Excluded from this study were those who had congenital malformations, prior ocular trauma, history of ocular surgery, ocular disorders except refractive errors, and who were incapable of cooperating.^29^ The study protocol was approved by the Ethics Committee Board of The Chinese University of Hong Kong. All children and their parents or guardians signed a written informed consent before their participation in the study. All study procedures adhered to the Declaration of Helsinki.

### Retinal Photography

We took fundus photographs of our participants throughout the day from 8.30 AM to 6 PM, after 30 minutes of rest. Retinal photographs were taken using a digital fundus camera (TRC-50DX; Topcon, Tokyo, Japan, with a color sensor resolution of 12.3 MP and image sensor of 1.1 inches) after pupil dilation using 1% cyclopentolate and 1% tropicamide under a standardized setting. Two retinal photographs, centered at the optic disc and the fovea, were obtained for each eye.

### Measurement of Retinal Vessel Caliber

The Singapore I Vessel Analyzer Deep Learning System (SIVA-DLS) is a fully automated AI Deep Learning System to measure retinal vessel caliber (see the Fig.).^28^ Prior to caliber estimation, initial gradability of the photograph was assessed by the SIVA-DLS, and photographs with poor image quality or unreliable caliber prediction by the SIVA-DLS were excluded from analysis.^28^ Photographs centered at the right eye optic disc were assessed. If the photograph was ungradable, measurements would be performed on the corresponding photograph for the left eye.^28^

**Figure.**
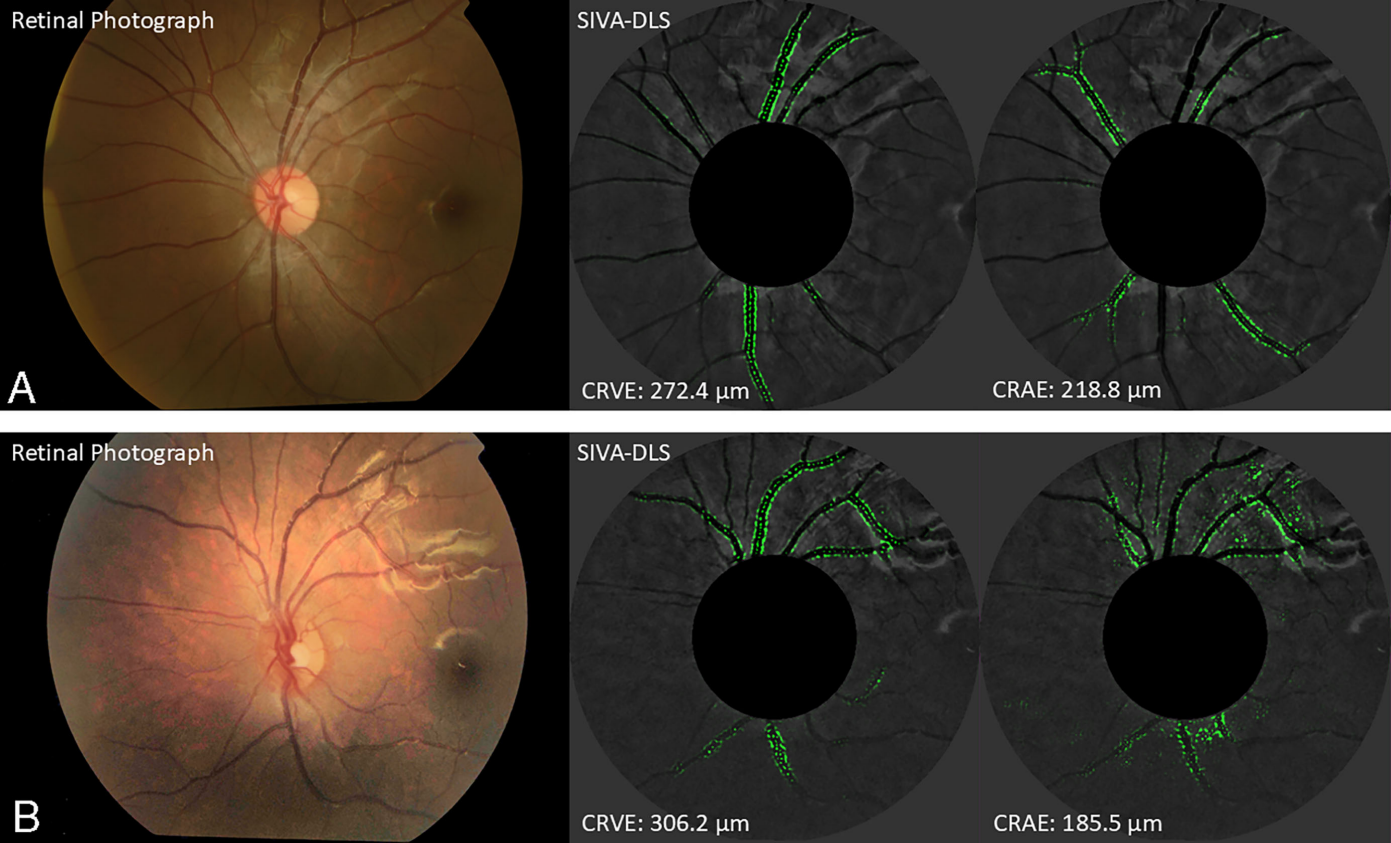
**Retinal photographs on the left, and SIVA-DLS heatmaps with venular and arteriolar boundaries highlighted to predict CRAE and CRVE on the right, of children more physically active (a) and less physically active (b).** The estimations of CRAE and CRVE computed by SIVA-DLS on retinal photographs of two children, one with higher ratio and the other with lower ratio. The heatmaps generated by SIVA-DLS highlighted the boundaries for arterioles and venules used to predict CRAE and CRVE, respectively.

The SIVA-DLS estimated the central retinal artery equivalent (CRAE), a summary index reflecting the average width of retinal arterioles, and the central retinal vein equivalent (CRVE), a summary index reflecting the average width of retinal venules from retinal photographs.^32^^,^^33^ It used a convoluted neural network to estimate the values in the region within 0.5 to 1.0 disc diameters away from the optic disc margin (Supplementary Materials).^28^ Heatmaps were generated to highlight the regions it focused on so as to calibrate its CRAE and CRVE predictions.^28^ After training and validation, the SIVA-DLS was externally tested using a large, multi-ethnic, multi-country dataset of >70,000 retinal photographs from 15 datasets, including one from the HKCES (see the Supplementary Materials).^28^

### Questionnaires

Parents or legal guardians of participating children were given questionnaires for information on demographics, socioeconomic status, and medical history of their children. The said questionnaires were derived from the Chinese version of questionnaires used in the Sydney Myopia Study,^34^^,^^35^ adjusted for cultural differences and local dialect by discussing with representatives of local teachers, parents, and ophthalmologists. They had been validated by a study on 100 children's parents to verify its reliability and validity.^29^ All data from the questionnaires were double entered to ensure integrity and precision, and parents would be further contacted for the completion of missing data in the questionnaires.

Selected questions from the questionnaires were used to assess physical activity and inactivity (see the Supplementary Materials). Parents were asked how long their children would engage in certain activities during weekdays, weekends, and holidays. Physical activity includes outdoor exercises, outdoor leisure activities, and indoor exercises, whereas physical inactivity includes watching television and digital video disc (DVD), playing video games, doing near work such as homework, reading, using a computer, and using other electronic devices.^26^^,^^36^ To reflect the overall activity level of children more comprehensively, we calculated the ratio of physical activity to inactivity (ratio) by dividing the total duration (hours per week) of physical activity by that of physical inactivity.

### Ophthalmological Examinations

Trained ophthalmologists conducted complete ocular examinations for each participant, including examinations of the anterior segment, posterior segment, and ocular motility. Refraction was measured before and after cycloplegia using an autorefractor (Nidek ARK-510A, Gamagori, Japan); and spherical equivalent refraction was calculated as the algebraic sum of the sphere value and half of the cylinder value. Ocular axial length was evaluated using an interferometric device (IOL Master; Carl Zeiss Meditec AG, Jena, Germany).

### Anthropometric Examination

Blood pressure (BP) was measured with the child in the seated position after a 5-minute rest using a digital autonomic BP monitor (Vital Signs Monitor; Heal Force Bio-Meditech, Shanghai, China), with an appropriate cuff size for accurate measurements. Three measurements were taken, and the averaged result was used for subsequent analysis. Body height and weight were measured using a professional integrated set (seca; Hamburg, Germany).

### Statistical Analysis

Statistical analysis was performed using SPSS, version 24 (version 24; IBM, Armonk, NY, USA). A *P* value of 0.05 was used to test for statistical significance. Descriptive statistics were presented as means (standard deviation). Skewness and kurtosis test for normality, Shapiro-Wilk test, and q-q plots were used to check assumptions of normality. Residual plots were inspected to check for the homogeneity of variance assumption. For continuous variables, the differences between their sexes were evaluated using the two-sample Student's *t-*test, whereas, for categorical data, the chi-squared test (χ²) was used. Linear regressions were performed and adjusted for sex, age, fellow vessel caliber, right eye axial length, weight, height, mean arterial pressure, and family income, similar to previous studies.^32^ The fellow vessel caliber was also adjusted for the analysis of CRAE and CRVE, so as to avoid biased results and minimize potential confounding from the fellow caliber.^37^ For example, CRVE would be added to the calculations of the effect of physical activity on CRAE, and vice versa.^37^ Standardized beta coefficients (*β*) with 95% confidence intervals (95% CIs) were used to estimate the effect size (difference in retinal vessel caliber, measured in µm).

## Results

### Study Participants

A total of 11,959 participants were included in the study, of which 6244 (52.22%) were boys; and the mean age was 7.55 years (SD = 1.047). The mean CRVE was 215.7 µm and the mean CRAE 151.6 µm. The mean physical activity (hours/week) was 18.74 and inactivity was 38.82. Boys had greater body mass index (BMI), height, weight, axial length, and shorter CRAE and CRVE than girls (Table 1).

**Table 1.** The Characteristics of the Study Participants

Characteristics	All Mean (SD)	Boys Mean (SD) (*n* = 6244)	Girls Mean (SD) (*n* = 5715)	*P* Value
Age, y	7.55 (1.05)	7.56 (1.02)	7.536 (1.07)	0.144
BMI, kg/m^2^	16.17 (3.07)	16.45 (3.25)	15.86 (2.82)	<0.001
Height, cm	124.88 (8.43)	125.37 (8.14)	124.34 (8.70)	<0.001
Weight, kg	25.49 (6.73)	26.14 (6.98)	24.78 (6.36)	<0.001
Mean arterial blood pressure, mm Hg	88.99 (14.93)	89.16 (14.12)	88.80 (15.77)	0.192
Axial length, mm	23.13 (0.93)	23.40 (0.90)	22.84 (0.87)	<0.001
Family income per mo, *N* (%)				
≤ HK $20,000^*^	3177 (26.57)	1646 (26.36)	1530 (26.77)	0.366
> HK $20,000^*^	8782 (73.43)	4598 (73.64)	4185 (73.23)	
Physical activity, h/wk	18.74 (3.22)	18.67 (3.22)	18.81 (3.21)	0.019
Physical inactivity, h/wk	38.82 (4.27)	38.84 (4.24)	38.80 (4.29)	0.661
Ratio	0.487 (0.091)	0.485 (0.091)	0.489 (0.092)	0.009
CRAE, µm	151.6 (11.5)	150.9 (11.3)	152.4 (11.6)	<0.001
CRVE, µm	215.7 (16.3)	214.9 (16.1)	216.6 (16.5)	<0.001

* Equivalent to US $2551.10.

### Associations of CRAE and CRVE With the Ratio of Physical Activity to Inactivity

Participants with increased ratio of physical activity to inactivity had wider CRAE (*β* = 1.033, 95% CI = 0.288 to 1.778, *P* = 0.007) and narrower CRVE (*β* = –2.079, 95% CI = –3.141 to –1.017, *P* < 0.001; Table 2), after adjusting for sex, age, fellow vessel caliber, right eye axial length, weight, height, mean arterial pressure, and family income.

**Table 2.** The Associations of CRAE and CRVE With the Ratio of Physical Activity to Physical Inactivity Across Different Models of Adjustment

		CRAE		CRVE	
		*β*-Coefficient (95% CI)	*P* Value	*β*-Coefficient (95% CI)	*P* Value
Ratio	Model 1	–0.270 (–1.656 to 1.116)	0.703	–2.412 (–4.386 to –0.437)	0.017
	Model 2	–0.483 (–1.860 to 0.895)	0.492	–2.615 (–4.579 to –0.651)	0.009
	Model 3	1.058 (0.311 to 1.805)	0.006	–2.037 (–3.102 to –0.971)	<0.001
	Model 4	1.031 (0.284 to 1.778)	0.007	–2.103 (–3.165 to –1.042)	<0.001
	Model 5	1.083 (0.341 to 1.825)	0.004	–2.162 (–3.218 to –1.105)	<0.001
	Model 6	1.060 (0.320 to 1.801)	0.005	–2.147 (–3.203 to –1.091)	<0.001
	Model 7	1.033 (0.288 to 1.778)	0.007	–2.079 (–3.141 to –1.017)	<0.001

Model 1: not adjusted.

Model 2: adjusted for sex and age.

Model 3: adjusted for sex, age, and fellow vessel caliber.

Model 4: adjusted for sex, age, fellow vessel caliber, and right eye axial length.

Model 5: adjusted for sex, age, fellow vessel caliber, right eye axial length, weight, and height.

Model 6: adjusted for sex, age, fellow vessel caliber, right eye axial length, weight, height, and mean arterial pressure.

Model 7: adjusted for sex, age, fellow vessel caliber, right eye axial length, weight, height, mean arterial pressure, and family income.

The subgroup analysis of boys showed that increased ratio was associated with wider CRAE (*β* = 1.364, 95% CI = 0.288 to 2.440, *P* = 0.013) and narrower CRVE (*β* = –2.563, 95% CI = –4.099 to –1.027, *P* = 0.001). In girls, the ratio was associated with narrower CRVE (*β* = –1.759, 95% CI = –3.237 to –0.282, *P* = 0.020), but not with CRAE (Table 3).

### Interactions of the Ratio of Physical Activity to Inactivity With Body Mass Index or Blood Pressure on CRAE and CRVE

There was no significant effect of the interactions of the ratio of physical activity to inactivity with body mass index on CRAE (*P* = 0.128) and CRVE (*P* = 0.120), as well as with BP on CRAE (*P* = 0.368) and CRVE (*P* = 0.844).

## Discussion

Physical activity improves cardiovascular health in children, which can be carried forward into adulthood,^2^^,^^38^ whereas physical inactivity can lead to overweight and obesity, which have been linked to cardiovascular diseases.^4^ Obese children are also five times more likely to be obese in adulthood,^5^ thus increasing the risk of noncommunicable diseases and premature mortality.^6^ During the pandemic, physical activity was much reduced while screen time and near work were substantially increased in children, contributing to increased ophthalmic complications and worsened physical health.^39^^–^^41^

Different techniques, both invasive and noninvasive, have been applied to investigate the microvasculature, from intracoronary vascular reactivity test in the heart, capillary microscopy in the skin, magnetic resonance imaging in the brain, to retinal imaging.^42^ As the microvasculature and systemic circulation share similar anatomic and physiological characteristics, previous studies have suggested that the microvasculature can be a marker of systemic vascular health, and that it can implicate cardiovascular, respiratory, renovascular, and neurovascular diseases.^18^^,^^43^^,^^44^ In the myocardium, increased physical activity is associated with angiogenesis and vasodilation^10^; whereas, in the skin, increased physical activity leads to greater heat‐induced skin hyperemia^45^^,^^46^; and, in skeletal muscles, physical activity increases regional blood flow capacity, capillary density, and arteriolar density.^47^ Although previous studies have investigated the effects of physical activity and inactivity on the retinal vasculature in adults,^23^^–^^25^^,^^48^ their effects in children were less explored and have been inconsistent.^26^^,^^49^ In addition, near work such as homework and reading also contributes to physical inactivity but was not analyzed in some studies.^50^ Moreover, physical activity and inactivity were often analyzed separately.^27^^,^^49^^,^^51^ However, this may not reflect the overall activity level comprehensively; as, for example, a child can have both high screen time and exercise time.

In this study, we showed that increased ratio of physical activity to inactivity, indicating an overall lifestyle of a more physically active nature, was associated with a healthier state of the retinal vasculature, as characterized by wider arterioles and narrower venules, which have been associated with improved cardiovascular health in adults.^52^ This reinforces the concept that physical activity has direct, measurable, and beneficial effects on the microvasculature in children.

Our study showed that physical inactivity alone did not have significant associations with CRAE and CRVE (Table 4). This is consistent with the previous studies in children by Imhof et al. and by Kochli et al.^49^^,^^50^ as well as the studies in adults by Tikellis et al. and Pressler et al.^23^^,^^53^ However, some previous studies reported inconsistent results between physical activity alone and retinal vessel caliber in children^26^^,^^50^ and in adults.^23^^,^^24^ This may be explained by the fact that physical activity and inactivity can be independent of each other, as a person's overall activity level should take both into account. For instance, a person can have a high level of both active time and inactive time; therefore, considering either parameter alone may not reflect the overall activity level and their combined effects on the retinal vasculature. Therefore, we included the ratio of physical activity to inactivity in our analysis. Our data showed that the higher the ratio, that is, more physically active overall, the wider CRAE and narrower CRVE, which were consistent with a number of previous studies.^26^^,^^51^

**Table 3.** The Associations of CRAE and CRVE With the Ratio of Physical Activity to Physical Inactivity in Boys, Girls, and all Participants

	CRAE		CRVE	
	*β*-Coefficient (95% CI)	*P* Value	*β*-Coefficient (95% CI)	*P* Value
Boys^*^	1.364 (0.288 to 2.440)	0.013	–2.563 (–4.099 to –1.027)	0.001
Girls^*^	0.877 (–0.161 to 1.916)	0.098	–1.759 (–3.237 to –0.282)	0.020
All participants^*^	1.033 (0.288, 1.778)	0.007	–2.079 (–3.141 to –1.017)	<0.001

* Adjusted for sex, age, fellow vessel caliber, right eye axial length, weight, height, mean arterial pressure, and family income.

**Table 4.** The Associations of CRAE and CRVE With Physical Activity, Physical Inactivity, and the Ratio of Physical Activity to Physical Inactivity

	CRAE		CRVE	
	*β*-Coefficient (95% CI)	*P* Value	*β*-Coefficient (95% CI)	*P* Value
Physical activity^*^	–0.018 (–0.032 to –0.005)	0.008	0.029 (0.010 to 0.048)	0.003
Physical inactivity^*^	0.004 (–0.026 to 0.033)	0.806	–0.019 (–0.061 to 0.023)	0.368
Ratio^*^	1.033 (0.288 to 1.778)	0.007	–2.079 (–3.141 to –1.017)	<0.001

* Adjusted for sex, age, fellow vessel caliber, right eye axial length, weight, height, mean arterial pressure, and family income.

The associations between physical activity and inactivity and the microvasculature may be explained by various mechanisms. Physical activity increases blood flow and shear stress, stimulating the release of nitric oxide, which can contribute to protection against cardiovascular diseases. Meanwhile, physical inactivity may result in an imbalance between nitric oxide and reactive oxygen species, leading to vascular dysfunction.^54^ In addition, physical activity has lipid-lowering and anti-inflammatory effects,^23^^,^^54^^,^^55^ which have been associated with changes in the microvasculature and cardiovascular health.

Being noninvasive and relatively accessible, retinal photography has been frequently investigated for cardiovascular disease monitoring, screening, and prevention in adults,^56^ with previous studies showing that adults with narrower arterioles are at increased risk for hypertension, incident stroke, coronary heart disease, and cardiovascular mortality.^57^ In children, changes in the retinal vasculature should also have important clinical implications, as these changes can be tracked to long term end-organ damage and mortality of cardiovascular diseases.^21^ First, changes in the retinal vasculature by physical inactivity in children may be reversible by treatment,^16^ suggesting that the retinal vasculature can be used for disease monitoring and treatment response. Second, changes in the retinal vasculature may occur early in the development of cardiovascular and metabolic diseases and thus may be used to predict their risks.^56^ In adults, it has been suggested that changes in CRAE and CRVE can be incorporated into existing prediction scores or used independently to predict cardiovascular diseases,^58^ and, therefore, a similar prediction score may also be developed in children.

Strengths of our study include a large, unselected, and population-based large sample of 11,959 children and the AI deep learning algorithm (SIVA-DLS) to automatically measure retinal vessel caliber from retinal photographs, which has been shown to perform comparably to expert graders, if not better in certain cases.^28^ Our study, however, is not without its limitations. First, we adopted a cross-sectional design which limited the exploration of casual and temporal relationships among retinal vessel caliber, physical activity, and inactivity. In this regard, a longitudinal approach can be used to improve the study, to assess whether changes in physical activity and health-promoting interventions can result in changes in retinal vessel parameters. Second, we utilized questionnaires in our study. Other assessments, such as shuttle run or sprinting, can also be included to assess physical activity. Physical activity and inactivity may also change over time, but questionnaires were only able to provide information at one timepoint only, not to mention its being also subjected to recall bias and social desirability bias. Third, there may be unmeasured confounding factors, including aerobic versus anaerobic exercises, intensity and vigor of physical activity, socioeconomic status, and environmental influences, such as circadian effects, nutritional status, sleep quality, stress, medications, allergies, and air pollution, which were not included in our study. Most of our participants were also ethnic Chinese. These factors may have the potential to impact the observed associations, and further studies are warranted to clarify these relationships.

## Conclusions

We demonstrated that increased physical activity in children is associated with ‟healthier” microvasculature, characterized by wider retinal arterioles and narrower venules. This has served to contribute to the growing evidence that physical activity positively influences vascular health from a young age, thus underscoring the potential of using the retinal vasculature as a biomarker of cardiovascular health.

## Supplementary Material

Supplement 1
