# Supplementary material for: Effects of Physical Activity and Inactivity on Microvasculature in Children: The Hong Kong Children Eye Study
Source: Invest Ophthalmol Vis Sci. 2024 Dec 3;65(14):7. doi: 10.1167/iovs.65.14.7 (PMC11620007; doi:10.1167/iovs.65.14.7)
Supplement: Supplement 1 [file iovs-65-14-7_s001.pdf]

Supplementary Materials

1. Specific questions in the questionnaire for measuring physical activity and inactivity

Translated English version:

Please indicate how long your child spends in the following activities each day on average. For example, if your child spends 15 hours in outdoor leisure activities from Monday to Friday, please indicate 3 hours (15 hours/5). If more than 3 hours on average, please indicate the exact number of hours.

Time (each day) / Categories	School day (For example: Monday to Friday)				Weekend （For example: Saturday and Sunday）				Holidays (For example: Summer holidays, Christmas)			
	Never	Less than 1 hour	1-2 hours	More than 3 hours	Never	Less than 1 hour	1-2 hours	More than 3 hours	Never	Less than 1 hour	1-2 hours	More than 3 hours
Out of doors （running, cycling, football, basketball, tennis, etc.）												
Outdoor leisure activities (BBQs, picnic, beach, bushwalk, etc.)												
Indoor exercise (gymnasium, dancing studio, indoor sports center, etc.)												
Watching T.V./ videos/ DVDs												
Playing video games (e.g. PlayStation)												

School homework												
Reading books for pleasure												
Using a computer												
Using electronic devices (phone, hand-held computer games, tablets)												

Original Chinese Version:

在下面的活動中，請選擇你的小朋友平均每日花在上面的時間（例如：如果你的小孩每個星期（星期一至星期五）參加戶外休閒活動15個小時，平均戶外休閒時間=15/5=3小時。）如果超過3個小時，請在横線上寫明具體時間

時間（每日） / 分類	**學習日（例如：週一至週五）**				**週末（例如：週六週日）**				**假期（例如：暑假，聖誕假）**			
	從不	少於1 小時	1-2小時	大於3小時	從不	少於1小時	1-2小時	大於3小時	從不	少於1小時	1-2小時	大於3小時
室外運動（跑步、踩單車、足球、室外籃球、網球等）												
戶外休閒活動(燒烤、野餐、海灘),被家長帶領出遊												

室內運動（健身房、舞蹈室、室內體育館等）												
看電視、 DVD												
玩電子遊戲機（如 PlayStation）												
完成學校的功課												
休閒閱讀												
用電腦												
玩電子產品（如：手機、手持遊戲機、平板電腦）												

2. Central retinal arteriolar equivalent and central retinal venular equivalent

Central retinal artery equivalent (CRAE) is a summary index reflecting the average width of retinal arterioles, and central retinal vein equivalent (CRVE) is a summary index reflecting the average width of retinal venules from retinal photographs. These measurements were made by using a modification of the Parr-Hubbard formulas[1] as described by Knudtson et al,[2] which showed clear superiority over the previous Parr-Hubbard formulas, providing more robust measurements that are independent of the number and the scale of retinal vessels measured.[3]

Modified formulas by Knudtson et al[2]

The modified formulas by Knudtson et al restricted the measurement of the retinal vessel diameters to the six largest retinal arterioles and venules measured from the photographs.

$$Arterioles: \widehat{W} = 0.88 * (w_1^2 + w_2^2)^{1/2}$$

$$Venules: \widehat{W} = 0.95 * (w_1^2 + w_2^2)^{1/2}$$

where W_1 is the width of the narrower branch, W_2 the width of the wider branch, and $\widehat{W}$ the estimate of parent trunk arteriole or venule.

Using these formulas, including only the six largest arterioles and the six largest venules, an iterative procedure was used to pair up the largest vessels with the smallest and repeating until a single number is reached, as a central vessel equivalent.

Parr-Hubbard formulas[1]

The Parr-Hubbard formulas measured the diameters of all arterioles and venules coursing through a concentric zone around the disc, then used the formula to combine pairs of branch measurements to obtain estimates of their trunks, and then combined pairs of trunk results in the same manner until all arterioles and venules had been summarized into a single central retinal artery equivalent (CRAE) and central retinal venular equivalent (CRVE).

$$Arterioles: W_c = 0.87\, {W_a}^2 + 1.01\, {W_b}^2 - 0.22\, W_a W_b - 10.76$$

$$Venules: W_c = 0.72\, {W_a}^2 + 0.91\, {W_b}^2 - 450.05$$

in which W_c is the caliber of the trunk vessel, W_a the caliber of the smaller branch, and W_b the caliber of the larger branch.

3. Singapore I Vessel Analyzer-Deep-Learning System[4]

Singapore I Vessel Analyzer-Deep-Learning System (SIVA-DLS) is a deep learning algorithm with convolutional neural networks for the automated measurement of retinal-vessel calibre in retinal photographs, developed from multiethnic multicounty datasets that comprise more than 70,000 images. The training and validation datasets included the datasets from

Singapore Chinese eye study (SCES), Singapore Indian eye study (SINDI), and Singapore Malay eye study (SiMES). The external testing datasets included the datasets from Singapore prospective study program (SP2), the Dunedin multidisciplinary health and development study (Dunedin study), Hong Kong children eye study (HKCES), Australian heart eye study (AHES), Retinal imaging in chest pain study (RICP study), Retinal imaging in renal disease study (IRED study), CUHK sight-threatening diabetic retinopathy study (CUHK-STDR study), Growing up in Singapore towards healthy outcomes birth cohort (GUSTO study), Singapore integrated diabetic retinopathy program (SiDRP), and Cardiovascular disease screening using retinal vascular imaging study (CVD screening study).

Performance of human graders and SIVA-DLS were independently tested. When compared between human graders and SIVA-DLS, agreement of calibre measurement in the validation and external testing datasets were good to excellent, with ICCs ranging from 0.82 to 0.95; agreement in individual external datasets was also good (all ICCs were above 0.7).

Multivariable linear regression analysis of cardiovascular disease risk factors and retinal-vessel calibre measured by SIVA-DLS and human graders were also tested. The associations of CRAE with age, gender, mean arterial blood pressure (MABP), body-mass index (BMI) and total cholesterol, and associations of CRVE with gender, MABP, BMI, glycated-haemoglobin and current smoking were largely identical between SIVA-DLS and human graders. For example, each s.d. increase in CRAE was associated with lower blood pressure (β, −3.67 mm Hg versus −3.28 mm Hg); and each s.d. increase in CRVE was associated with higher glycated-haemoglobin level (β, 0.312% versus 0.295%), comparing SIVA-DLS versus SIVA-human, respectively.

4. Collinearity analysis

We performed collinearity analysis to investigate the collinearity between variables. All variables had variance inflation factor (VIF) less than 5, indicating no significant collinearity.

CRAE

Variables	Variance Inflation Factor (VIF)
Sex	1.120
Age	2.061
Fellow vessel caliber (CRVE)	1.053
Right eye axial length	1.352
Weight	2.051
Height	3.056
Mean arterial pressure	1.065
Family income	1.013
Ratio of physical activity to inactivity	1.015

CRVE

Variables	Variance Inflation Factor
Sex	1.120
Age	2.062

Fellow vessel caliber (CRAE)	1.054
Right eye axial length	1.342
Weight	2.055
Height	3.056
Mean arterial pressure	1.067
Family income	1.013
Ratio of physical activity to inactivity	1.015

References

1. Hubbard LD, Brothers RJ, King WN, et al. Methods for evaluation of retinal microvascular abnormalities associated with hypertension/sclerosis in the Atherosclerosis Risk in Communities Study. *Ophthalmology* 1999;106:2269-2280.
2. Knudtson MD, Lee KE, Hubbard LD, Wong TY, Klein R, Klein BE. Revised formulas for summarizing retinal vessel diameters. *Curr Eye Res* 2003;27:143-149.
3. Sun C, Liew G, Wang JJ, et al. Retinal Vascular Caliber, Blood Pressure, and Cardiovascular Risk Factors in an Asian Population: The Singapore Malay Eye Study. *Investigative Ophthalmology & Visual Science* 2008;49:1784-1790.
4. Cheung CY, Xu D, Cheng C-Y, et al. A deep-learning system for the assessment of cardiovascular disease risk via the measurement of retinal-vessel calibre. *Nature Biomedical Engineering* 2021;5:498-508.
